# Impaired IFN-γ-mediated innate and adaptive immunity in Coffin-Siris syndrome type 2: immunological insights from a patient with a recurrent *ARID1A* mutation

**DOI:** 10.3389/fimmu.2026.1803570

**Published:** 2026-07-09

**Authors:** Qi Peng, Yi Yang, Yaozhong Zhang, Siping Li, Baimao Zhong, Qingming Luo, Xiaomei Lu

**Affiliations:** 1 Laboratory Department, Dongguan Children’s Hospital Affiliated to Guangdong Medical University, Dongguan, Guangdong, China; 2 Department of Medical and Molecular Genetics, Dongguan Institute of Pediatrics, Dongguan, Guangdong, China; 3 Key Laboratory for Children’s Genetics and Infectious Diseases of Dongguan, Dongguan, Guangdong, China; 4 Department of Pediatrics, Dongguan Maternal and Child Health Care Hospital, Dongguan, Guangdong, China

**Keywords:** ARID1A, chromatin remodeling, Coffin-Siris syndrome, immunodeficiency, innate immunity, interferon-gamma, transcriptional response

## Abstract

**Background:**

Coffin-Siris syndrome type 2 (CSS2), caused by *ARID1A* mutations, is characterized by neurodevelopmental delay and recurrent infections. The *ARID1A* p.Ala1077Glu variant has been linked to CSS2, but the immune mechanisms underlying infection susceptibility remain poorly understood.

**Methods:**

The variant was identified by trio-based whole-exome sequencing and confirmed by Sanger sequencing. Immune status was assessed via serum immunoglobulins, complement measurements, and flow cytometric immunophenotyping. RNA sequencing was performed on PMA-stimulated PBMCs from the patient and healthy controls, with key findings validated by qRT-PCR and ELISA.

**Results:**

Immune profiling revealed widespread quantitative deficits across innate and adaptive compartments, with particularly marked reductions in NKT-like cells (CD3^+^CD56^+^) and CD8^+^ central memory T cells. Transcriptomic analysis of stimulated cells uncovered a profound activation defect, characterized by widespread downregulation of effector immune genes. Furthermore, pathway enrichment analysis demonstrated significant suppression of multiple biological processes critically dependent on IFN-γ signaling, such as antigen processing and presentation, and Th1/Th17 cell differentiation. Expression of *IFNG*, *STAT1*, and *CXCL9* showed a trend toward decreased expression.

**Conclusion:**

Our findings suggest an association between the ARID1A p.Ala1077Glu variant and impaired IFN-γ-mediated immunity in CSS2, potentially contributing to recurrent infections. The IFN-γ pathway warrants further investigation as a therapeutic target.

## Introduction

Coffin-Siris syndrome (CSS, OMIM#135900) is a rare neurodevelopmental disorder classically characterized by intellectual disability, distinctive coarse facial features, and anomalies of the fifth digit (1). The phenotypic spectrum has expanded over time, with recurrent infections now recognized as a prominent feature observed in approximately 60% of patients (2). However, the immune dysfunction underlying this susceptibility remains poorly defined.

CSS is caused by heterozygous pathogenic variants in 14 known genes, including *ARID1A, ARID1B, ARID2, SMARCA2, SMARCA4, SMARCB1, SMARCC2* and others (3–5). Most of these genes encode subunits of the BRG1/BRM-associated factor (BAF) chromatin remodeling complex (6–8). Pathogenic variants in *ARID1A* cause CSS type 2 (CSS2) by disrupting chromatin accessibility and transcriptional regulation (9, 10). Chromatin remodelers function as central regulators in the immune system by establishing lineage-specific chromatin accessibility (11–13). Whether germline mutations in these regulators predispose to specific immunodeficiencies in CSS remains underexplored.

The *ARID1A* variant NM_006015.6:c.3230C>A (p.Ala1077Glu) was initially reported in a patient with CSS2 presenting with neurodevelopmental manifestations, recurrent respiratory infections, and immune thrombocytopenia (10). This established the variant’s association with CSS2, yet its specific mechanistic impact on human immunity remains uncharacterized. In mice, myeloid-specific *Arid1a* deletion impairs type I interferon production and antiviral defense (14). Whether *Arid1a* similarly regulates the type II interferon (IFN-γ) pathway is less explored. In cancer, somatic ARID1A loss suppresses IFN-γ signaling (15). However, the consequences of germline ARID1A dysfunction on human immune competence remain unknown.

Here, we report a second, unrelated individual with CSS2 carrying the same germline *ARID1A* p.Ala1077Glu variant, with severe recurrent pneumonia. To test the hypothesis that this variant induces immune dysregulation, we performed deep immune phenotyping and transcriptomic analysis of stimulated PBMCs. Our findings suggest an impairment in IFN-γ-mediated signaling that may contribute to immune dysfunction in CSS2.

## Materials and methods

### Human subjects and ethics

This study was approved by the Ethics Committee of Dongguan Children’s Hospital Affiliated to Guangdong Medical University (Approval No. LL2022121502). Written informed consent was obtained from the parents/legal guardians. The proband, born in September 2019, presented with features consistent with CSS. Three age- and sex-matched healthy children served as controls.

### Genetic analysis

Genomic DNA was extracted from peripheral blood using the QIAamp DNA Blood Mini Kit (Qiagen, Hilden, Germany). Trio-based whole-exome sequencing (WES) was performed by Jiajian Medical Laboratory (Guangzhou, China) using the Illumina NovaSeq 6000 platform. Sequence data were processed, annotated, and filtered following standard bioinformatic pipelines. The identified variant was further validated by Sanger sequencing. The pathogenicity of the variant was assessed in accordance with the American College of Medical Genetics and Genomics and the Association for Molecular Pathology (ACMG/AMP) guidelines.

### Comprehensive immune analysis

Humoral immunity and complement system activity, levels of serum immunoglobulins (IgA, IgG, IgM) and complement components (C3, C4), along with κ- and λ-light chains, were measured.

High-dimensional flow cytometric immunophenotyping of peripheral blood was performed at 3 years and 6 months of age by Guangzhou KingMed Centre for Clinical Laboratory Co., Ltd., a CAP- and ISO15189-accredited laboratory. This assay quantified absolute counts and relative frequencies of leukocyte subsets, monocyte/dendritic cell populations, and lymphocyte differentiation/activation states.

### PBMC isolation, PMA stimulation, and RNA extraction

PBMCs were isolated from freshly collected EDTA-anticoagulated peripheral blood (10 mL) within 2 hours using density-gradient centrifugation over lymphocyte separation medium. After centrifugation at 500 × g for 30 minutes, the PBMC layer was harvested, washed twice with PBS (250 × g, 10 min), and resuspended in RPMI-1640 with 10% FBS. PBMCs from the proband and controls were stimulated with 100 nM PMA (Sigma-Aldrich) for 3 hours at 37 °C in 5% CO_2_. Total RNA was extracted using the RNeasy Plus Mini Kit (Qiagen).

### RNA sequencing and bioinformatic analysis

RNA-Seq was performed on PBMCs from the patient (n = 1) and age- and sex-matched healthy controls (n = 3). Total RNA was extracted using identical protocols. RNA quality was evaluated across all samples (RIN scores in **Supplementary Table 3**).

Paired-end (2×150 bp) sequencing was performed on the DNBSEQ platform. All samples were processed in a single batch to minimize technical variation. Reads were aligned to GRCh38 using STAR (v2.7.10a), and gene expression was quantified as read counts.

Given the exploratory single-patient design, genes with |log_2_FC| ≥ 1.5 and nominal p < 0.05 (prior to multiple-testing correction) were selected for KEGG pathway enrichment. FDR was not used as a filtering criterion. Pathways with nominal p < 0.05 were considered hypothesis-generating trends.

### Quantitative real-time PCR

Quantitative real-time PCR (qRT-PCR) was performed on a LightCycler^®^ 480 II instrument (Roche Diagnostics, Switzerland) using the EZB 2×Colour SYBR Green qPCR Master Mix (EZBioscience, China). PBMCs from the patient and a matched healthy control were assayed in technical triplicate. Target gene expression was normalized to GAPDH and calculated using the 2^(–ΔΔCt) method.

### Measurement of IFN-γ protein expression by ELISA

IFN-γ concentration in serum and culture supernatants was measured using an ELISA kit (Thermo Fisher Scientific, Cat. No. BMS228). The assay sensitivity was 0.99 pg/mL. ELISA was performed per the manufacturer’s protocol, with optical density read at 450 nm. Sample concentrations were interpolated from a standard curve. Assays compared the patient (n = 1) to the matched control (n = 1).

### Statistical analysis

Statistical analysis and graphing were performed using GraphPad Prism. Due to the single-patient design, qRT-PCR and ELISA results are reported descriptively without formal statistical inference.

## Results

### Clinical presentation of a CSS2 patient with recurrent infections

**Figure 1:**
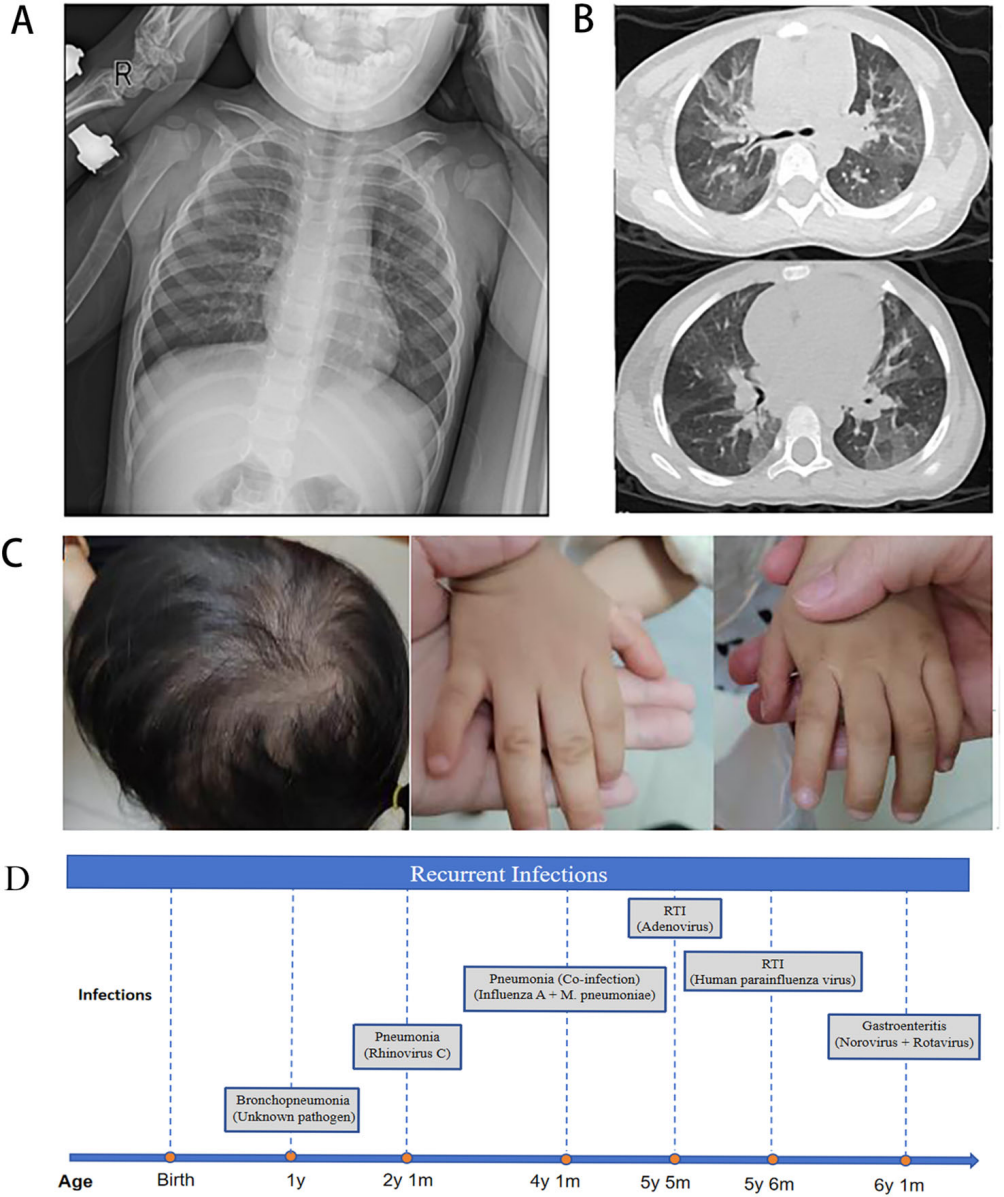
Clinical and radiologic findings in a CSS2 patient with recurrent infections. **(A)** Chest radiograph at presentation, showing findings consistent with bronchopneumonia. **(B)** Chest computed tomography (CT) scan demonstrating patchy opacities, bronchial wall thickening, and airway narrowing, indicative of bronchopneumonia with underlying airway involvement. **(C)** Photographs of the proband, illustrating key dysmorphic features including sparse scalp hair and brachydactyly (short distal phalanges). **(D)** Clinical timeline of infectious episodes confirmed at our institution over the four-year follow-up period. Each icon represents a documented infection event. This timeline captures only severe episodes requiring hospital evaluation and does not reflect the total infectious burden, which includes milder upper respiratory infections managed at home or at other facilities.

The female proband first presented to our institution at 2 years of age with a persistent cough lasting over 20 days. Chest radiography confirmed bronchopneumonia (**Figure 1A**), and computed tomography (CT) revealed heterogeneous lung attenuation with a mosaic pattern, scattered patchy opacities, thickened bronchovascular bundles, and bronchial wall thickening with luminal narrowing, indicating underlying airway involvement (**Figure 1B**).

Her history included chronic constipation, strabismus, and global developmental delay, with limited speech and independent walking achieved at 23 months. Examination revealed failure to thrive (weight and height between -1SD and -2SD) and dysmorphic features including abnormal facies, nystagmus, perineal vitiligo, sparse hair, and brachydactyly (**Figure 1C**). The severe respiratory episode at age 2 occurred within an ongoing pattern of recurrent infections. Based on caregiver reports, the patient experienced an estimated 8–9 upper respiratory infections annually over the prior 12 months, with nearly half reportedly progressing to pneumonia. Only those infections confirmed at our institution over the four-year follow-up are shown (**Figure 1D**); milder episodes or those managed elsewhere are not captured.

### Genetic diagnosis and ARID1A variant identification

**Figure 2:**
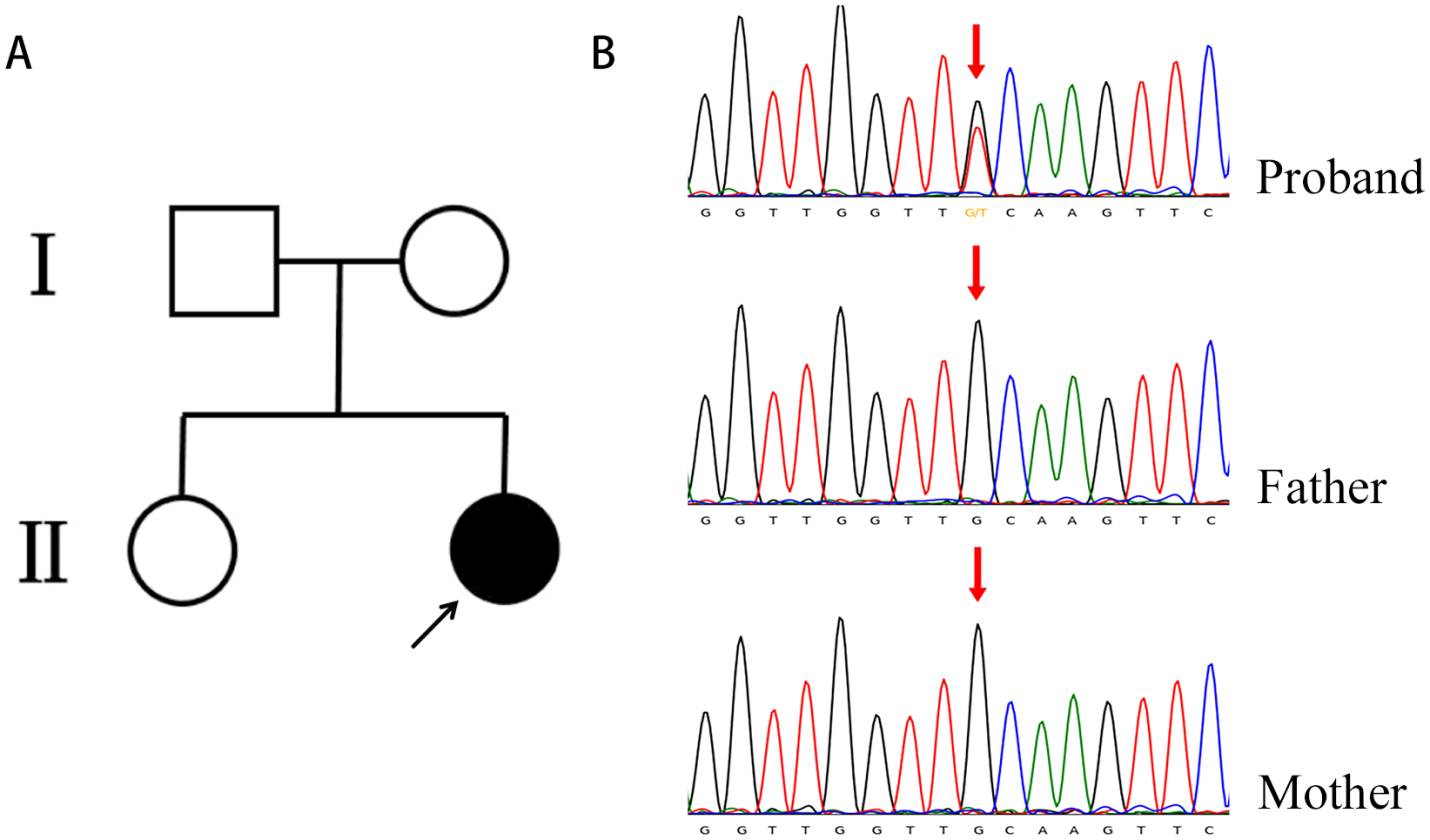
Identification of a *de novo ARID1A* variant and molecular diagnosis of CSS2. **(A)** Pedigree of the proband (indicated by arrow) and her parents. **(B)** Sanger sequencing chromatograms confirming the heterozygous *de novo* missense variant c.3230C>A (p.Ala1077Glu) in the proband’s *ARID1A* gene.

Trio-based whole-exome sequencing revealed a heterozygous missense variant, c.3230C>A (p.Ala1077Glu), in the proband’s *ARID1A* gene, absent in both parents (**Figure 2A**). Sanger sequencing further confirmed the variant and verified its *de novo* origin (**Figure 2B**).

According to the ACMG/AMP guidelines, this variant meets the criteria for a likely pathogenic classification (PS2, PS4_Supporting, PM2_Supporting, PP3), confirming the diagnosis of CSS2. Detailed descriptions and justifications for each applied ACMG/AMP criterion are summarized in **Supplementary Table 1**.

### Comprehensive immunoprofiling uncovers widespread dysregulation across innate and adaptive compartments

Humoral immunity assessment (immunoglobulins and complement) was unremarkable (**Table 1**). High-dimensional flow cytometric immunophenotyping was performed. Total lymphocyte counts were normal, but profiling revealed widespread dysregulation across multiple compartments (**Table 2**). Full immunophenotyping data, including antibody panels and reference ranges, are in **Supplementary Table 2**.

**Table 1:** Serum immunoglobulin and complement levels of the patient.

Parameters	Measured value	Reference interval
Immunoglobulin A (IgA)	0.36 g/L	0.14–1.14
Immunoglobulin G (IgG)	8.43 g/L	3.82–10.58
Immunoglobulin M (IgM)	0.82 g/L	0.40–1.28
Complement C3	0.93 g/L	0.79–1.52
Complement C4	0.27 g/L	0.16–0.38
κ-light chain	6.97 g/L	6.29–13.50
λ-light chain	3.93 g/L	3.13–7.23
κ/λ ratio	1.77	1.47–2.95

**Table 2:** Summary of abnormal immune function indicators by functional category.

Functional module	Abnormal indicator (cell/subset)	Value	Unit	Flag	Reference Range
Innate Immunity	Total Leukocytes (WBC)	12,740	/μL	↑	4850 – 11640
	Pro-inflammatory Monocytes	0.77%	%	↓	2.20 – 16.70%
	(CD14^+^CD16^high^)	6.58	/μL	↓	7.00 – 86.00
	NKT Cells (CD3^+^CD56^+^)	0.50	%	↓	8.6-15.9%
Adaptive Immunity (T Cells)	Double Negative T Cells (DNT)	6.04%	%	↑	0.56 – 2.36%
		169.93	/μL	↑	16.00 – 58.00
	CD8^+^ Central Memory T Cells	4.56%	%	↓	8.10 - 34.30%
		35.43	/μL	↓	54.00 – 379.00
	CD8^+^ Effector Memory T Cells	12.34	%	↑	0.60 – 12.01%
Regulatory Immunity	Naive Regulatory T Cells (Naive Treg)	0.00%	%	↓	3.50 – 77.30%
		0.00	/μL	↓	4.00 – 68.00
	Memory Regulatory T Cells (Memory Treg)	100.00%	%	↑	22.70 – 96.50%
		152.76	/μL	↑	6.00 – 118.00
	CD4^+^CD27_−_ Anergic T Cells	1.42	%	↓	1.50 – 36.90%
	CD4^+^CD57^+^ Senescent T Cells	0.20	%	↓	0.60 – 30.40%
Mucosal/Tissue Immunity	TCR γδ1 T Cells	50.24	%	↑	1.00 – 30.00%
	TCR γδ2 T Cells	27.65	%	↓	50.00 – 90.00%
Humoral Immunity (B Cells)	Transitional B Cells	4.49	%	↓	4.73 – 15.68%
	Plasmablasts	0.06%	%	↓	0.60 – 10.31%
		0.66	/μL	↓	4.00 – 88.00
	CD21low B Cells	1.79	%	↓	1.90 – 19.30%
	Non-switched Memory B Cells (abs.)	33.91	/μL	↑	1.00 – 28.00

↑ means above reference range; ↓ means below reference range.

Notable innate findings included leukocytosis and markedly reduced NKT-like cells (CD3^+^CD56^+^). Adaptive defects included expanded DNT and altered CD8^+^ T cell differentiation, with decreased central memory and increased effector memory subsets. The Treg pool was profoundly imbalanced, completely skewed toward a memory phenotype with absent naive Tregs. TCR γδ T cell subsets showed a significant shift, and B-cell differentiation was impaired, with markedly reduced plasmablasts.

### Transcriptomic analysis of stimulated PBMCs uncovers global suppression of immune pathways

RNA-seq was performed on PMA-stimulated PBMCs from the patient and healthy controls. The estimated common biological coefficient of variation (BCV) was 0.177, within the typical range for human PBMC transcriptomic studies (0.1–0.3). While acknowledging the inherent uncertainty due to the small control group (n=3), this estimate suggests no evidence of excessive technical or biological variability among the control samples.

After filtering for expressed genes (CPM ≥ 1 in at least two samples), 12,739 genes were retained from an initial 17,063 genes. Using the exploratory criteria |log_2_FC| ≥ 1.5 and nominal p < 0.05, we identified 115 differentially expressed genes. FDR values were not used as a filtering criterion. Of these, 92 genes were annotated by DAVID and subjected to KEGG pathway enrichment analysis. Quality control metrics (read counts, Q30 scores, mapping rates) are in **Supplementary Table 3**; full DEG lists and KEGG results are in **Supplementary Tables 4** and ** 5**.

**Figure 3:**
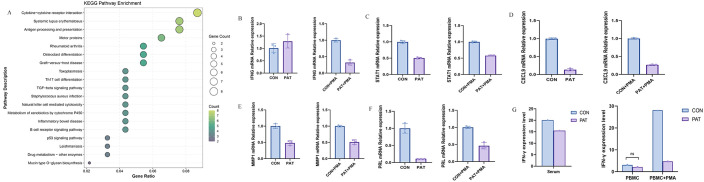
Transcriptomic and functional analyses indicate a blunted immune response and attenuated IFN-γ signaling in the patient. **(A)** Kyoto Encyclopedia of Genes and Genomes (KEGG) pathway enrichment analysis of downregulated differentially expressed genes (DEGs) in PMA-stimulated peripheral blood mononuclear cells (PBMCs) from the patient compared to healthy controls. Significance is determined based on nominal p < 0.05, as justified by the single-patient study design. **(B–F)** Validation of the transcriptional trend in the IFN-γ signaling axis. qRT-PCR analysis showing mRNA expression levels in PMA-stimulated PBMCs from the patient (n = 1) versus an age- and sex-matched healthy control (n = 1). Bars represent mean ± SD of technical triplicates. Data are presented descriptively; no statistical inference was performed. **(G)** ELISA quantification of IFN-γ. Left panel (Serum): CON vs. PAT. Right panel (PBMC and PBMC+PMA): CON vs. PAT under unstimulated and PMA-stimulated conditions. Baseline levels were comparable; PMA-stimulated IFN-γ secretion was blunted in the patient. Data are descriptive (n = 1 per group); no statistical inference.

KEGG pathway enrichment was performed on the 92 annotated DEGs. Given the single-patient design, enrichment was evaluated using nominal p-values (p < 0.05) rather than adjusted p-values, to capture potentially relevant biological trends without overly stringent penalization. This analysis revealed a trend of downregulation across multiple pathways associated with inflammation and immune responses (**Figure 3A**), including “Cytokine-cytokine receptor interaction”, “Natural killer cell mediated cytotoxicity” (consistent with the NKT-like cell deficiency identified by flow cytometry), and “Th17 cell differentiation”. The “Antigen processing and presentation” pathway was also downregulated, but was largely driven by KIR family members (e.g., *KIR3DS1, KIR2DL5A*), whose expression is restricted to NK/NKT cells. Given the marked reduction in NKT-like cells (**Table 2**), this downregulation most likely reflects the depletion of this cell population rather than a cell-intrinsic transcriptional defect.

The most pronounced downregulation among all detected transcripts was CCL3L1 (log_2_FC = -16.03), a chemokine abundantly expressed by NKT cells. Given the near-complete absence of NKT-like cells by flow cytometry (**Table 2**), this reduction is consistent with depletion of this population rather than a cell-intrinsic transcriptional defect. This pattern of widespread downregulation suggests an attenuated transcriptional response in the patient’s PMA-stimulated cells, consistent with the clinical phenotype of recurrent infections.

### The IFN-γ signaling axis is prominently affected

To validate the transcriptomic findings, we focused on the IFN-γ signaling axis. Genes for qRT-PCR validation were selected based on biological relevance to IFN-γ signaling rather than fold-change magnitude alone; for example, *STAT1* was included despite its modest RNA-seq fold-change. Consistent with RNA-seq, qRT-PCR showed decreased mRNA expression of *IFNG* and its key downstream targets (*STAT1*, *CXCL9*), as well as MMP1 and PRL, in the patient’s PMA-stimulated PBMCs compared to the matched healthy control (**Figures 3B–F**). This transcriptional pattern was accompanied by an attenuated protein secretory response. Notably, while baseline serum IFN-γ concentrations and levels in unstimulated PBMC culture supernatants were comparable between the patient and the control, enzyme-linked immunosorbent assay (ELISA) detected a blunted secretion of IFN-γ protein in the patient’s PBMC culture supernatants following PMA stimulation (**Figure 3G**).

## Discussion

Our integrated analysis identifies a previously uncharacterized immune phenotype in CSS2, suggesting an association between the recurrent *ARID1A* p.Ala1077Glu variant and blunted IFN-γ-mediated immunity. By bridging deep immunophenotyping, transcriptomic profiling, and functional validation, we suggest that dysregulated chromatin function may contribute to infection susceptibility—a hypothesis that warrants further investigation.

IFN-γ is a master regulator of innate and cell-mediated immunity, essential for macrophage activation, Th1 differentiation, and pathogen clearance (16–18). The consistent downregulation of IFN-γ signaling at both the mRNA and protein levels toward a functional defect, providing a potential link to the patient’s recurrent pneumonia. This finding aligns with emerging evidence: somatic *ARID1A* loss in cancer silences IFN-γ-responsive genes (15), while myeloid- specific *Arid1a* deletion in mice impairs antiviral responses (14). Our work extends these observations to a human germline *ARID1A* mutation, suggesting that CSS2 may be positioned within the expanding spectrum of chromatinopathy-associated immunodeficiencies.

The immune dysregulation likely reflects both contributors to and consequences of impaired IFN-γ signaling. NKT-like cells—potent early producers of IFN-γ (19)—were markedly reduced, a deficiency that could compromise early pathogen containment (20). Defective IFN-γ signaling may also impair CD8^+^ central memory T cell maintenance (21), and the near-absence of plasmablasts suggests broader B-cell dysregulation potentially driven by a disrupted cytokine milieu. These alterations may represent a combination of direct effects of *ARID1A* dysfunction on immune development and secondary adaptations to chronic IFN-γ signaling impairment.

When interpreting transcriptomic data, a distinction should be made between signals reflecting altered cell composition and those indicating cell-intrinsic defects. The marked downregulation of *CCL3L1* and the KIR-driven enrichment of the “Antigen processing and presentation” pathway are both consistent with NKT-like cell depletion by flow cytometry. In contrast, the core IFN-γ signaling components—*IFNG*, *STAT1*, and *CXCL9*—are broadly expressed across multiple immune lineages. Their consistent downregulation suggests that the IFN-γ signaling defect may extend beyond NKT-cell depletion and could involve broader, potentially cell-intrinsic, transcriptional impairment.

Mechanistically, we hypothesize that the germline *ARID1A* p.Ala1077Glu variant may create a transcriptional bottleneck at critical immune gene loci, though direct evidence of altered chromatin accessibility is not yet available. Importantly, because PMA bypasses surface receptors to directly activate protein kinase C (PKC), the patient’s blunted response suggests that this bottleneck lies downstream of cytosolic signaling. As *ARID1A* normally facilitates nucleosome displacement, the failure to mount an adequate transcriptional response despite potent PKC activation raises the possibility that the mutated cBAF complex fails to efficiently remodel chromatin or facilitate transcriptional elongation, particularly at IFN-γ-responsive promoters. We emphasize that this mechanistic model remains a hypothesis; direct testing—such as ATAC-seq to assess chromatin accessibility or functional rescue experiments in patient-derived cells, will be required in future studies.

Our study has several limitations. First, the use of bulk PBMCs cannot exclude the possibility that transcriptomic differences are partially driven by compositional shifts; single-cell or sorted-subset analyses are needed to distinguish cell-intrinsic defects from compositional effects. Second, as a single-patient study, the generalizability of this IFN-γ-centric defect requires validation in larger, genotyped cohorts. Third, PMA is a non-physiological stimulus; future studies using pathogen-associated stimuli (e.g., TLR agonists) will better recapitulate *in vivo* challenges. Fourth, our study infers but does not directly demonstrate altered chromatin dynamics; ATAC-seq on patient-derived cells or iPSC-differentiated lineages is required to test whether the variant impairs chromatin accessibility at IFN-γ-responsive promoters. Fifth, our KEGG enrichment relied on nominal p < 0.05 without FDR correction; therefore, the pathway trends in **Figure 3A** are hypothesis-generating observations only and should not be interpreted as statistically significant.

## Conclusion

In conclusion, this study identifies a clinically relevant immune phenotype in a patient with a germline ARID1A missense variant, consistent with impaired IFN-γ-mediated immunity in CSS2. Our findings suggest a potential molecular basis for the observed infection susceptibility, though the precise mechanisms remain to be defined. The IFN-γ axis emerges as a prominent feature of immune dysregulation in this context and warrants exploration as a therapeutic target for severe cases. This work suggests a link between a chromatin remodeling defect and a specific innate immune deficiency in a human developmental disorder.

## Data Availability

The datasets generated and analysed during the current study are available in public repositories. The genetic variant data are available in the ClinVar repository (accession number SCV002540197). The RNA-seq data are available in the Sequence Read Archive (SRA) repository (accession number PRJNA1447406).
