# Supplementary material for: Impaired IFN-γ-mediated innate and adaptive immunity in Coffin-Siris syndrome type 2: immunological insights from a patient with a recurrent ARID1A mutation
Source: Front Immunol. 2026 Jul 9;17:1803570. doi: 10.3389/fimmu.2026.1803570 (PMC13391312; doi:10.3389/fimmu.2026.1803570)
Supplement: Supplementary file 1 [file Table1.docx]

| **ACMG Code** | **Description** |
| --- | --- |
| PS2 | De novo (both maternity and paternity confirmed) in our patient with the disease and no family history. |
| PS4_Supporting | The same missense variant (c.3230C>A, p.Ala1077Glu) has been identified in two unrelated probands with Coffin-Siris syndrome type 2 and consistent core phenotypes (developmental delay, coarse facial features, brachydactyly, recurrent infections): one previously reported by Liu et al. (2022, PMID: 35353340) and the patient described in the present study. Per ClinGen guidelines for ultra-rare disorders, this observation supports PS4_Supporting (rather than full PS4) in the absence of formal case-control statistical analysis. |
| PM2_Supporting | Absent from controls in gnomAD v4.1.1, as well as in the Exome Sequencing Project, 1000 Genomes, and ExAC. |
| PP3 | Multiple in silico tools consistently predict a deleterious effect: AlphaMissense (1.0, pathogenic), REVEL (0.74, pathogenic), CADD (27, deleterious), MetaRNN (0.90, pathogenic), PrimateAI (0.89, pathogenic). |

**Table S1. ACMG/AMP Evidence Classification for the ARID1A c.3230C>A (p.Ala1077Glu) Variant**
